# Regional Homogeneity Predicts Creative Insight: A Resting-State fMRI Study

**DOI:** 10.3389/fnhum.2018.00210

**Published:** 2018-05-23

**Authors:** Jiabao Lin, Xuan Cui, Xiaoying Dai, Lei Mo

**Affiliations:** ^1^Center for Studies of Psychological Application, Guangdong Key Laboratory of Mental Health and Cognitive Science, School of Psychology, South China Normal University, Guangzhou, China; ^2^Guangdong Provincial Key Laboratory of Mental Health and Cognitive Science, South China Normal University, Guangzhou, China

**Keywords:** creative insight, resting-state fMRI, chunk decomposition, neural correlate, regional homogeneity

## Abstract

Creative insight plays an important role in our daily life. Previous studies have investigated the neural correlates of creative insight by functional magnetic resonance imaging (fMRI), however, the intrinsic resting-state brain activity associated with creative insight is still unclear. In the present study, we used regional homogeneity (ReHo) as an index in resting-state fMRI (rs-fMRI) to identify brain regions involved in individual differences in creative insight, which was compued by the response time (RT) of creative Chinese character chunk decomposition. The findings indicated that ReHo in the anterior cingulate cortex (ACC)/caudate nucleus (CN) and angular gyrus (AG)/superior temporal gyrus (STG)/inferior parietal lobe (IPL) negatively predicted creative insight. Furthermore, these findings suggested that spontaneous brain activity in multiple regions related to breaking and establishing mental sets, goal-directed solutions exploring, shifting attention, forming new associations and emotion experience contributes to creative insight. In conclusion, the present study provides new evidence to further understand the cognitive processing and neural correlates of creative insight.

## Introduction

Creative insight is generally defined as a sudden comprehension that lead to a new interpretation of a situation and yield the solution to a problem (Sternberg and Davidson, 1995; Kounios et al., 2006; Kounios and Beeman, 2009; Webb et al., 2016). Studies of creative insight have been conducted for more than a dozen years. For example, previous studies have investigated the characteristics of creative insight, including influencing factors (Öllinger et al., 2008; Yang et al., 2016), phases (Amabile, 1988), and cognitive mechanism (Kershaw and Ohlsson, 2004). However, as a specific form of creative thinking, the precise neural correlates of creative insight remain unknown.

Previous studies have conjectured that creative insight could involve in several principle components: emotional experience, breaking and establishing mental sets, and reorienting attention. For example, several studies indicated that participants experienced positive feelings in the process of insight problem-solving (Jung-Beeman et al., 2004; Ludmer et al., 2011). Problem solvers would encounter an impasse if their initial mental representations of the problem were incorrect, and they were required to break such impasses and restructure the initial representation during the problem-solving (Ohlsson, 1992; Knoblich et al., 1999). Moreover, Tang et al. (2016) showed that preventing irrelevant objects from being attended and goal-directed attentional orienting were needed in the experience of creative insight. Thus, creative insight might recruit widespread processing in the cognitive control networks, attentional networks and emotion systems. However, the specific brain regions in these networks underlying the creative insight are still unclear.

Several neuroimaging studies have explored the neural correlates of creative insight with task-based functional magnetic resonance imaging (fMRI; Luo and Niki, 2003; Jung-Beeman et al., 2004; Subramaniam et al., 2009; Zhao et al., 2013; Huang et al., 2018). For instance, Jung-Beeman et al. (2004) used a type of problem called compound remote associates (CRA) to investigate the neural correlates of creative insight. The CRA problem offered three words (e.g., pine, crab, sauce), and participants were required to come up with a single word (e.g., apple) that can form compound phrases (e.g., pineapple, crabapple and applesauce) with each of the three problem words. The author found that insight solutions were associated with brain activity in superior temporal gyrus (STG). With the similar task, Subramaniam et al. (2009) detected that anterior cingulate cortex (ACC) showed sensitivity to creative insight. Moreover, using a specific form of insight problem-solving, called riddles solving, Zhao et al. (2013) detected that the insight condition activated the region of ACC. And another studies showed that temporoparietal junction (TPJ) activations were probably associated with the insightful riddle-solving (Luo and Niki, 2003; Huang et al., 2018). In summary, these findings suggested that the ACC, STG and TPJ may be related to creative insight in a particular task. However, task-related fMRI studies have an obvious drawback that a particular task only activates particular regions (Xiang et al., 2016). And the neural mechanisms of individuals with tendency to experience insight across a variety of tasks were still uncleared. Thus, it was necessarily to elucidate the intrinsic brain mechanisms of creative insight. In this study, we mainly focused on the linkage between the insightful problem-solving and the resting-state brain activity.

Recently, a method of resting-state fMRI (rs-fMRI), regional homogeneity (ReHo), has been developed to analyze the brain activity in normal participants and patients. The ReHo measures the ReHo of the spontaneous blood oxygenation level dependent (BOLD) fluctuations during rest (Zang et al., 2004). In addition, the ReHo targets on the local synchronization of voxels within a specific brain area, which is different from the functional connectivity focused on the long-distance interregional temporal correlations of BOLD signals. Previous studies have confirmed that ReHo was an effective index to reflect the neural activity of resting state in a variety of domains, including intelligence (Wang et al., 2011), human emotion (Xiang et al., 2016), healthy aging (Wu et al., 2007), Parkinson’s Disease (Wu T. et al., 2009) and Alzheimer’s disease (He et al., 2007). Therefore, in the current study, we used this method to investigate brain neural activity of creative insight in the resting state.

Chunk decomposition, defined as decomposing familiar patterns into basic elements so that they can be reorganized in a novel way, is a specific form of insightful problem-solving and reflects feature of human creativity (Scott, 1962; Ohlsson, 1992; Knoblich et al., 2001; Öllinger et al., 2008; Wu L. et al., 2009; Nijstad et al., 2010; Dreu et al., 2011; Huang et al., 2015; Tang et al., 2016). In this study, we used a revised chunk decomposition task, called Chinese character chunk decomposition, to calculate the degree of creative insight in each participant, and further explore the neural mechanisms of creative insight. First, we designed two types of creative Chinese character chunk decomposition: creative chunk decomposition-low level (CCDL) and creative chunk decomposition-high level (CCDH). The CCDL was defined as requiring participants to transform a Chinese character into a new one by removing a particular part, such as a stroke, which was novel to participants but could be solved with a high probability of success. While in the CCDH condition, participants were asked to transform a character into another one by removing an intact character, like an isolated character, which was much more novel to subjects and could be solved with a low probability of success. Second, for each participant, we calculated the mean response time (RT) by averaging the RT of the CCDL condition and CCDH condition. Then we used this mean RT to represent the creative insight score of each participant. Longer (shorter) RT indicated lower (higher) creative insight score. Finally, we computed the correlation between creative insight and ReHo across the whole brain to see which brain regions were related to creative insight.

In this rs-fMRI study, based on the concept of creative insight and the findings of previous task-fMRI studies (Luo and Niki, 2003; Jung-Beeman et al., 2004; Subramaniam et al., 2009; Zhao et al., 2013; Huang et al., 2018), we hypothesized that individual differences in creative insight would be significantly correlated with the ReHo in some brain regions like the ACC, STG and TPJ. Because these brain regions are mainly linked to conflict monitoring, breaking and establishing mental sets, reorienting attention and affective function (Ellison et al., 2004; Fan et al., 2007; Radua et al., 2012; Chang et al., 2013).

## Materials and Methods

### Participants

A total of 50 right-handed and paid volunteers (23 M/27 F, aged 18–27 years old, 21.26 ± 2.13 years old) were included in this study. Participants were healthy, had normal or corrected-to-normal vision, and reported no history of neurological or psychiatric disorders. This study was carried out in accordance with the recommendations of University’s Policy for Ethical Practice, Research Ethics Review Board of South China Normal University. The protocol was approved by the Research Ethics Review Board of South China Normal University. All subjects gave written informed consent in accordance with the Declaration of Helsinki.

### Materials

**Figure 1:**
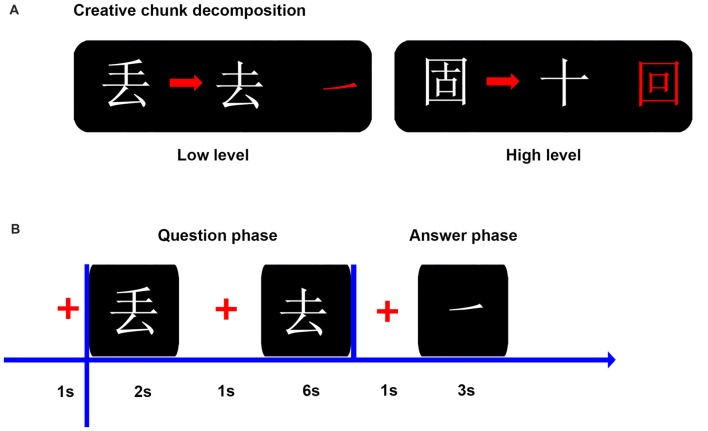
Illustration of the experiment materials and procedure. **(A)** Examples of two experimental conditions: creative Chinese character chunk decomposition-low level; creative Chinese character chunk decomposition-high level. Both conditions were defined by operational definitions (i.e., spatial relationship between the target character and the to-be-removed part or character) and novelty assessment. The to-be-removed part and character are also showed in red color on the right side. **(B)** The flowchart shows the time course of a trial in behavior experiment. In the question phase, participants were asked to determine the to-be-removed portion (a part or an intact character), from transforming the existing character into the target character as soon as possible. In the answer phase, the answer portion (a part or an intact character) was presented and participants were requested to confirm whether it was the same as the to-be-removed portion found in the question phase as soon as possible.

Chinese characters are ideal examples of chunks because of its orthographic structure (Tan et al., 2001). Previous studies have confirmed the validity of using Chinese character chunk decomposition to investigate the insight problem-solving (Luo et al., 2006; Tang et al., 2016). In this study, two types of creative Chinese character chunk decomposition, including CCDL and CCDH, were created, based on both operational definition and novelty assessment by a separate sample of 20 participants on a 2-point scale (Figure 1). Specifically, the CCDL condition required participants to transform a character into a new one by removing a specific part (e.g., a stroke), which was novel to participants but could be solved with a high probability of success. In the meanwhile, the CCDH condition asked participants to transform a character into a new one by removing an intact character (e.g., an isolated character), which was much more novel to participants and could be solved with a low probability of success. Novelty assessment by paired-samples *t*-test showed that there was significant difference in novelty between the CCDL condition and CCDH condition (*t*_(1,19)_ = −14.09, *p* < 0.001). All Chinese characters selected as experimental materials in this study are frequently used characters.

### Behavioral Task and Procedure

We designed two experimental conditions in this study: CCDH and CCDL (Figure 1). Each condition included 80 trials, and the total 160 trials were equally divided into two blocks, with a block consisting of 40 trials per condition. The order of the two blocks was counterbalanced across participants and trials were randomized in each block. Participants were asked to decompose parts or characters from existing characters to constitute new characters (target characters). The experiment took place in a sound-attenuated booth. Participants sat in front of the computer screen with their hands located in front of the keyboard. The computer screen resolution was 1024 * 768. The distance between the participant’s head and the screen was about 70 cm.

In each trial, the character need to be decomposed was presented on the screen for a fixed duration of 2 s and participants were asked to remember this character. After a 1 s fixation cross, another character (target character) was shown on the screen, and participants had 6 s to determine the to-be-removed portion (a part or an intact character) by pressing a button as soon as possible, from decomposing the to-be-decomposed character into the target character. Then, after a 1 s fixation cross, participants had to decide whether the portion found previously was the same as the portion that appeared on the subsequent screen within 3 s by pressing a button as soon as possible. Before the experiment, participants performed a training with different stimuli than those used for the formal experiment (Figure 1).

After the behavioral experiment, all participants then underwent an MRI scan during which they were instructed to refrain from head movement and remain awake. The scan was comprised of anatomical imaging (5 min) and resting state imaging (8 min).

### Creative Insight Assessment

Previous studies have verified that chunk decomposition is a specific form of insightful problem-solving (Scott, 1962; Ohlsson, 1992; Knoblich et al., 2001; Öllinger et al., 2008; Wu L. et al., 2009; Nijstad et al., 2010; Dreu et al., 2011; Huang et al., 2015; Tang et al., 2016). In the present study, we adopted a revised Chinese character chunk decomposition task to assess the degree of creative insight in each participant. Specifically, for each participant, we first calculated the mean RT by averaging the RT of CCDL condition and the RT of CCDH condition. Then we used this mean RT to represent the creative insight score of each participant. Longer (shorter) RT indicated lower (higher) creative insight score. Previous studies have confirmed that it was reasonable to use RT as an index of reflecting creative insight (Wu et al., 2013; Huang et al., 2015).

### MRI Data Acquisition

All images were acquired on a 3T Siemens Trio Tim MRI scanner in South China Normal University. For each participant, the rs-fMRI data, consisted of 240 images (8 min), was acquired using a gradient-echo-planar imaging (EPI) sequence with the following parameters: repetition time (TR) = 2000 ms, echo time (TE) = 30 ms, thickness = 3.5 mm, field of view (FOV) = 204 × 204 mm^2^, flip angle (FA) = 90°, data matrix = 64 × 64, and 33 axial slices covering the whole brain. In addition, the high-resolution brain structural image was obtained using a T1-weighted 3D magnetization prepared rapid acquisition gradient echo (MP-RAGE) sequence with the following parameters: TR = 1900 ms, TE = 2.52 ms, FA = 9°, data matrix = 256 × 256, FOV = 256 × 256 mm^2^, thickness = 1.0 mm, and 176 sagittal slices covering the whole brain. During the rs-fMRI scan, each participant was requested to relax, close eyes and wake, but not thinking about other things.

### MRI Data Preprocessing

The images were preprocessed using SPM8^1^ and DPABI^2^ (Yan and Zang, 2010). For each participant, the first 10 functional volumes were discarded to allow for scanner equilibration. The remaining 230 images were corrected by slice-timing and realigned for head motion correction. Then, the realigned images were co-registered with the T1-weighted image and normalized to a voxel size of 3 × 3 × 3 mm^3^, using a standard Montreal Neurological Institute (MNI) template. At last, we performed signal linear detrending, band-pass filtering (0.01–0.08 Hz), and regressed out the nuisance covariates, including head motion parameters derived from the Friston 24-parameter model, white matter signal, and cerebrospinal fluid signal within each voxel in whole brain. And we have not regressed out the global signal in this study. We estimated 6-parameter head motion and all of subjects satisfied our criteria: translation <2.5 mm in any plane and angular rotation <2.5° in any direction during realignment.

### Calculation of ReHo

We calculated the ReHo using the DPABI toolbox. Briefly, Kendall’s coefficient of concordance (KCC; Kendall and Gibbons, 1990) was used to measure ReHo or similarity of the time series of a given voxel with its nearest 26 neighbor voxels in a voxel-wise manner. The formula used to calculate the KCC has been reported elsewhere (Zang et al., 2004). Then, an individual KCC map was obtained for each participant. For standardization purposes, each individual ReHo map was divided by the global mean ReHo to reduce the influence of individual variations in the KCC value across the participants. Finally, the images were smoothed with a 4 mm full width at half maximum (FWHM) Gaussian kernel to reduce noise and residual differences in gyral anatomy. In addition, a previous study indicated that the standardization step of dividing ReHo of each voxel by the mean whole brain ReHo would reduce the variance of the ReHo values of this voxel across participants (Wang et al., 2011). Due to this controversy, we also examined the relationships between the original ReHo maps and the creative insight to supplement the findings in the present study.

### Statistical Analyses

In behavior data analysis, a paired-samples *t*-test was used to assess the differences in novelty between the CCDL condition and CCDH condition, using SPSS software (version 19.0; SPSS, Chicago, IL, USA) and significant level was set at *p* < 0.01 (two-sided). In the rs-fMRI whole-brain analyses, a multiple linear regression was conducted by using creative insight (defined by mean RT) as the variable of interest to identify regions where ReHo was correlated with individual differences in the level of creative insight after controlling for possible confounding variables, such as age and sex. We determined the surviving clusters at a voxel level threshold of *p* < 0.005 (uncorrected) and cluster level threshold of *p* < 0.05 (FWE corrected) to correct for multiple comparisons.

### Test-Reproducibility Procedure

A previous study indicated that the magnitude used for spatial smoothing during preprocessing stage or the numbers of nearest voxels to be measured might affect the KCC value (Zang et al., 2004). To ensure the reproducibility of the main findings in this study, we smoothed the images with a 6 mm FWHM Gaussian kernel to investigate the relationship between the ReHo and the creative insight. And we also measured the ReHo of a given voxel with its nearest 18 neighbor voxels in a voxel-wise manner and studied the linkage between the ReHo and the creative insight. Finally, we determined whether our main findings in the present study could be reproduced or not. We also determined the surviving clusters at a voxel level threshold of *p* < 0.005 (uncorrected) and cluster level threshold of *p* < 0.05 (FWE corrected) to correct for multiple comparisons.

## Results

**Figure 2:**
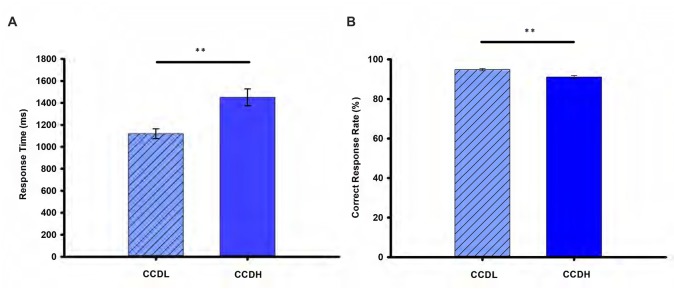
Behavioral results. Panel **(A)** shows the mean response time (RT) and **(B)** shows the mean correct response rate (%) in the creative chunk decomposition-low level (CCDL) and creative chunk decomposition-high level (CCDH). Error bars correspond to the standard error. The horizontal cap lines with “**” represent *p*-value < 0.01.

Figure 2 showed details of the mean RT and correct rate (CR) of each experimental condition. Specifically, paired-samples *t*-test revealed that RT of CCDL was significantly longer than that of CCDH (*t*_(49)_ = −7.92, *p* < 0.001). Moreover, CR of CCDL was significantly higher than that of CCDH (*t*_(49)_ = 4.51, *p* < 0.001). The behavioral data confirmed our predicted hypothesis.

**Figure 3:**
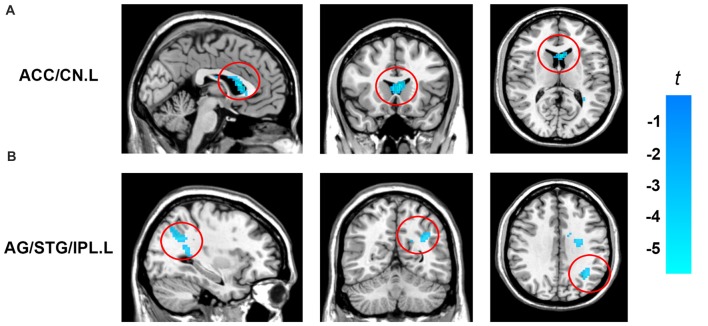
Brain regions showing significant correlation with creative insight. Activation maps are shown at a voxel level threshold of *p* < 0.005 (uncorrected), and cluster level threshold of *p* < 0.05 (FWE corrected). Images are plotted with the MRIcron (https://www.nitrc.org/projects/mricron). **(A)** ReHo of the left ACC/CN was negatively correlated with the creative insight. **(B)** ReHo of the left AG/STG/IPL showed negatively correlated with the creative insight. Abbreviations: ReHo, regional homogeneity; STG, superior temporal gyrus; ACC, anterior cingulate cortex; CN, caudate nucleus; AG, angular gyrus; IPL, inferior parietal lobe.

**Figure 4:**
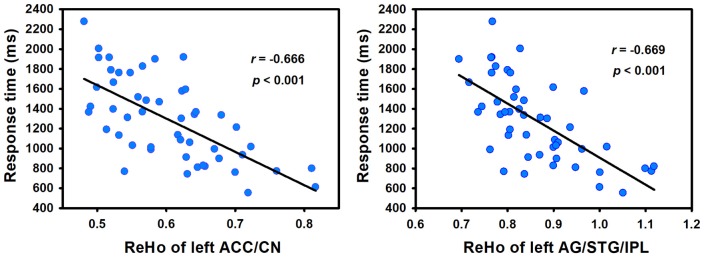
Creative insight scores (defined by the mean RT of both creative chunks decomposition) showed negative correlation with ReHo in the left ACC/CN and left AG/STG/IPL. Abbreviations: ReHo, regional homogeneity; STG, superior temporal gyrus; ACC, anterior cingulate cortex; CN, caudate nucleus; AG, angular gyrus; IPL, inferior parietal lobe.

In order to determine the neural correlates of creative insight, we correlated the creative insight score with the ReHo value of each voxel across the whole brain to explore the neural correlates of creative insight. After controlling for age and sex, the creative insight scores were significantly and negatively associated with two clusters located in the left ACC/caudate nucleus (CN), and left angular gyrus (AG)/STG/inferior parietal lobe (IPL). Details of these clusters were showed on Table 1, Figures 3, 4. In addition, we examined the relationships between the original ReHo maps and the creative insight using the same whole-brain analysis. The results showed that no clusters were survived after multiple comparison corrections.

**Table 1:** Brain regions showing significant correlation with creative insight.

Regions	Side	Cluster size (voxels)	MNI coordinates			*t* value
			*x*	*y*	*z*	
ACC/CN	LH	182	−3	18	12	5.75
AG/STG/IPL	LH	114	−33	−57	33	4.56

*Clusters were obtained at a voxel level threshold of p < 0.005 (uncorrected) and cluster level threshold of p < 0.05 (FWE corrected) to correct for multiple comparisons. Coordinates are the stereotactic space of the Montreal Neurological Institute. The t value corresponds to the peak voxel showing greatest statistical difference within a cluster. Abbreviations: STG, superior temporal gyrus; ACC, anterior cingulate cortex; AG, angular gyrus; CN, caudate nucleus; IPL, inferior parietal lobe; LH, left hemisphere*.

To assess the reproducibility of the results, we smoothed the images with a 6 mm FWHM Gaussian kernel during the preprocessing stage and further calculated the relationship between the ReHo and the creative insight. In addition, we measured the ReHo of a given voxel with its nearest 18 neighbor voxels and then studied the relationship between the ReHo and the creative insight. The results of both analyses revealed that ReHo in the left ACC/CN and left AG/STG/IPL were significantly and negatively correlated with creative insight, thus replicating the results obtained in the present study (Table 2).

**Table 2:** Brain regions revealing significant correlation with creative insight in the test-reproducibility study.

Regions	Side	Cluster size (voxels)	MNI coordinates			*t* value
			*x*	*y*	*z*	
FWHM = 6 mm during the preprocessing stage			
ACC/CN	LH	214	−3	18	9	5.56
AG/STG/IPL	LH	154	−33	−57	33	4.57
KCC calculation (numbers of nearest voxels = 18)			
ACC/CN	LH	135	−3	18	12	5.44
AG/STG/IPL	LH	73	−33	−60	33	4.61

*Clusters were obtained at a voxel level threshold of p < 0.005 (uncorrected) and cluster level threshold of p < 0.05 (FWE corrected) to correct for multiple comparisons. Coordinates are the stereotactic space of the Montreal Neurological Institute. The t value corresponds to the peak voxel showing greatest statistical difference within a cluster. Abbreviations: FWHM, full width at half maximum; KCC, Kendall’s coefficient of concordance; STG, superior temporal gyrus; ACC, anterior cingulate cortex; AG, angular gyrus; CN, caudate nucleus; IPL, inferior parietal lobe; LH, left hemisphere*.

## Discussion

To the best of our knowledge, this is the first study that used the ReHo method and chunk decomposition paradigm to explore the potential contribution of spontaneous brain activity to creative insight. The results showed that creative insight scores were negatively correlated with ReHo in the left ACC/CN and left AG/STG/IPL, suggesting that these brain regions were associated with creative insight. These results were proved to be stable and repeatable in a reproducibility test.

We observed creative insight scores were significantly and negatively correlated with ReHo in the ACC/CN. Previous studies indicated that the ACC was related to conflict detection, cognitive adaptation, goal-directed behavior, and affective functions (Botvinick et al., 1999; Fan et al., 2007; Radua et al., 2012; Huang et al., 2015). And the CN was the core region of the procedural memory system (Barnes et al., 2005; Seger, 2006), and could be related to the habit formation (Graybiel, 2008), novelty assessment (Tang et al., 2016) and sequence learning (Boyd et al., 2009). In addition, previous studies have indicated that the creative insight involved in inhibiting the predominant irrelevant mental representations, establishing new representation in a goal-directed way, and positive emotional experience (Bowden and Jung-Beeman, 2003; Subramaniam et al., 2009). For instance, Bowden and Jung-Beeman (2003) found that problem solvers experienced their solutions of insightful problems as sudden and surprising, which called the “*Aha!* moment”. Subramaniam et al. (2009) emphasized that problem solvers were required to detect competing solution candidates, rely less on dominant associations, and break and establish set to solve the insight problem. Creative insight included processes to break mental sets, assess the novelty of the insightful solutions, and form new task-related associations (Bowden and Jung-Beeman, 2007; Tang et al., 2016). Thus, the finding that ReHo in the ACC/CN predicted creative insight may suggest a relationship between creative insight and conflict monitoring, breaking and establishing mental sets, novel information storage and emotional experience. Furthermore, previous task-fMRI studied showed that ACC and CN was activated in insight problem-solving, which was partially consistent with our current study (Luo et al., 2004; Subramaniam et al., 2009; Zhao et al., 2013; Huang et al., 2015).

In addition to the ACC/CN, creative insight was also significantly and negatively correlated with ReHo in the cluster of AG/STG/IPL. The AG was an association area related to manipulate mental representations, reasoning and comprehension (Seghier et al., 2010; Seghier, 2013). The STG played a critical role in representing spatial awareness, and object-related exploration (Karnath, 2001; Ellison et al., 2004). And the IPL was involved in shifting attention, intention understanding and sustaining attention on task goals (Fogassi et al., 2005; Singh-Curry and Husain, 2009). Moreover, it was worth noting that the AG/STG/IPL belongs to the TPJ, which was mainly responsible for reorienting attention (Chang et al., 2013), updating mental expectations (Murray et al., 2015), and perspective taking (Santiesteban et al., 2012). Furthermore, previous studies about creative insight indicated that solvers would come to an impasse when trying to solve an insight problem, and would produce the solution after a variety of solution attempts (Dominowski and Dallob, 1995; Bowden and Jung-Beeman, 2003). In task-related fMRI studies of creative insight, using the similar task of Chinese character chunk decomposition, Tang et al. (2016) found that activated IPL may support preventing irrelevant objects from being attended in the experience of creative insight. And with the task of solving riddles, Huang et al. (2018) indicated that TPJ was involved in finding an appropriate solution to the insight problem and restructuring the initial mental representations to goal states. In addition, the TPJ was found to be extensively activated during creative generation and evaluation (Ellamil et al., 2012). These findings indicated that insight problem solving required more efforts to explore solutions and shift attentions. In summary, the result that ReHo in the AG/STG/IPL or the TPJ predicted creative insight significantly may indicate a relationship between creative insight and object-related exploration, shifting attention, reasoning, and acting in goal-directed way. However, further research was needed to explore this possibility, since the explanation of the above activated brain regions was based on findings from previous studies.

In addition, the IPL was a core region associated with the default mode network (DMN) in humans (Raichle et al., 2001; Buckner et al., 2008). The brain regions in the DMN were typically deactivated during the performance of goal-directed tasks (Raichle et al., 2001; Greicius et al., 2003). Previous studies indicated that the higher the level of cognitive load, the more deactivation in the DMN was observed (Mayer et al., 2010; Preminger et al., 2011). In the present study, we detected that the ReHo in the IPL was negatively associated with the creative insight score, which indicated that the DMN may play an important role in insight problem-solving.

In the present study, we used the ReHo as a measure of the regional coherence of the spontaneous BOLD fluctuations during rest. As stated by Zang et al. (2004), ReHo reflected the synchronization of activity in different brain regions, and it could be modulated in pertinent cognitive tasks. Fox et al. (2005) suggested that neuronal synchrony may efficiently organize the information processing of the brain. Moreover, previous studies indicated that ReHo reflected the strength of local connectivity within a cluster (Paakki et al., 2010; Wang et al., 2011). From this perspective, we suggested that creative insight may modulate the neuronal synchrony in the brain regions of left ACC/CN and left AG/STG/IPL, and decreased local connectivity in these brain regions may contribute to the high creative insight in the individuals.

Finally, we sketched a model of creative insight which defines the functional role of each brain region. We speculated that the activations in the AG/STG/IPL or TPJ involved in exploring, shifting attention, and looking for the insightful solution which is meaningful and target-related at the same time. The ACC/CN were related to inhibiting the predominant irrelevant task representations, restructuring the representations in a goal-directed way, and established the novel representations. Moreover, the activations in the ACC could be partly related to the “Aha!” experience accompanied with insight problem solving, and CN played an important role in the evaluation of novel solutions and forming novel associations in creative insight.

### Limitations and Future Directions

This study has several limitations. First, in the present study, we used the mean RT of both chunk decompositions as an index to represent the degree of creative insight, which was controversial. This approach was reasonable to some extent since higher creative insight score indicated more difficulty. And previous studies of chunk decomposition have confirmed that the insightful solution often accompanied longer RT than the ordinary solution, and RT could be an index partly reflecting the extent of insight (Huang et al., 2015; Tang et al., 2016). However, the RT might simply reflect the task difficulty, not insight itself. Thus, in the study, we also used the mean CR to characterize the degree of creative insight. Higher (lower) CR indicated higher (lower) creative insight score. However, results of the whole brain analysis showed that there were no significant correlations between the ReHo and the creative insight. In addition, we also measured the relationship between CR and the regions significantly correlated with RT by means of ReHo. Results showed that only the cluster of AG/STG/IPL was significantly and positively correlated with creative insight (*r* = 0.291, *p* = 0.040; Supplementary Figure S1). It seems that RT might be a more sensitive index of creative insight than CR. In the future, a more accurate index or task is needed to characterize the creative insight. Second, we noticed that several studies indicated the hippocampus (HIP) could be involved in creative insight, which was contradicted with the present study (Luo and Niki, 2003; Zhao et al., 2013). In these riddle-solving studies, the authors defined insight as presentation of the answer to which participants indicated they could understand the meaning of the answer. We speculated that the activation in the HIP could be result from retrieving existing memory to comprehend the answer of riddles, not insight itself. Further research was needed to explore this possibility in future. Third, in the present study, we determined the surviving clusters at a voxel level threshold of *p* < 0.005 (uncorrected) and cluster level threshold of *p* < 0.05 (FWE corrected) to correct for multiple comparisons, which might increase the rate of false positive. To supplement our findings, we used a threshold of cluster size for multiple comparison correction. The 3dClustSim program was used to correct for multiple comparisons in AFNI (10,000 iterations). The smoothing kernel was calculated with 3dFWHMx. The new reestimated size of spatial smoothness was larger than original (original: 4, 4, 4 mm; new: 9.45, 9.41 and 9.22 mm). Using the new smooth size for multiple comparison correction, the voxel-wise intensity threshold was set at *p* < 0.005, and a cluster threshold of *p* < 0.05 (Cluster size ≥70) was set. Results showed that only the cluster of left ACC/CN survived at such a threshold of cluster size for multiple comparison correction. In addition, we set the voxel level threshold of *p* < 0.001 and the clusters involved more than 30 voxels were reported, all of the results in the present study were replicated although each corresponding cluster decreased a little in cluster size (Supplementary Table S1). Finally, in this study, we showed the roles of several brain regions in creative insight. However, the relationships between these regions, and the brain networks underlying the creative insight were still unclear. In future, studies including functional connectivity and structural connectivity are needed to reveal the neural correlates of creative insight.

## Conclusion

With the rs-fMRI and chunk decomposition paradigm, the present study provides the new evidence that spontaneous brain activity is linked to creative insight. The ReHo in the ACC/CN, and the AG/STG/IPL can be used to predict individual differences in creative insight. Furthermore, the ACC/CN are implicated in emotional experience, conflict monitoring, breaking mental sets, and forming task-related associations. The AG/STG/IPL involves in shifting attention, reasoning, and looking for insightful solutions. In brief, the present study contributes to further understanding the cognitive processing and neural correlates of creative insight.

## Author Contributions

JL contributed to the experimental design, data analysis and writing of the initial manuscript. LM contributed to revising the experimental design and manuscript. XC and XD coordinated data collection.

## Conflict of Interest Statement

The authors declare that the research was conducted in the absence of any commercial or financial relationships that could be construed as a potential conflict of interest.

## Footnotes

^1^http://www.fil.ion.ucl.ac.uk/spm/

^2^http://rfmri.org/dpabi

## Abbreviations

ACC: anterior cingulate cortex
AG: angular gyrus
BOLD: blood oxygenation level dependent
CCDH: creative chunk decomposition-high level
CCDL: creative chunk decomposition-low level
CN: caudate nucleus
CR: correct rate
CRA: compound remote associate
DMN: default mode network
fMRI: functional magnetic resonance imaging
FWHM: full width at half maximum
HIP: hippocampus
IPL: inferior parietal lobe
KCC: Kendall’s coefficient of concordance
MNI: Montreal Neurological Institute
ReHo: regional homogeneity
rs-fMRI: resting-state fMRI
RT: response time
STG: superior temporal gyrus
TPJ: temporoparietal junction.
