# Supplementary material for: Regional Homogeneity Predicts Creative Insight: A Resting-State fMRI Study
Source: Front Hum Neurosci. 2018 May 23;12:210. doi: 10.3389/fnhum.2018.00210 (PMC5974035; doi:10.3389/fnhum.2018.00210)
Supplement: Supplementary file 1 [file Data_Sheet_1.docx]

Table S1. Brain regions showing significant correlation with creative insight based on two types of reporting standards. (A) Clusters were obtained at a voxel level threshold of *p* < 0.005 (uncorrected) and cluster level threshold of *p* < 0.05 (FWE corrected) to correct for multiple comparisons. (B) We set the voxel level threshold of *p* < 0.001 and the clusters involved more than 30 voxels were reported. Coordinates are the stereotactic space of the Montreal Neurological Institute. The *t* value corresponds to the peak voxel showing greatest statistical difference within a cluster. Abbreviations: STG, superior temporal gyrus; ACC, anterior cingulate cortex; AG, angular gyrus; CN, caudate nucleus; IPL, inferior parietal lobe; LH, left hemisphere.

| Regions | Side | Cluster size | MNI coordinates | | | *t* value |
| --- | --- | --- | --- | --- | --- | --- |
|  |  | (voxels) | x | y | z |  |
| (A) Voxel-level *p* < 0.005 (uncorrected) and cluster-level *p* < 0.05 (FWE corrected) | | | | | | |
| ACC/CN | LH | 182 | -3 | 18 | 12 | 5.75 |
| AG/STG/IPL | LH | 114 | -33 | -57 | 33 | 4.56 |
| (B) Voxel-level *p* < 0.001 (uncorrected) and clusters involved more than 30 voxels | | | | | | |
| ACC/CN | LH | 74 | -3 | 18 | 12 | 5.75 |
| AG/STG/IPL | LH | 32 | -33 | -57 | 33 | 4.56 |
