# Supplementary material for: Regional Homogeneity Predicts Creative Insight: A Resting-State fMRI Study
Source: Front Hum Neurosci. 2018 May 23;12:210. doi: 10.3389/fnhum.2018.00210 (PMC5974035; doi:10.3389/fnhum.2018.00210)
Supplement: Supplementary file 2 [file Data_Sheet_2.docx]

Figure S1. Creative insight scores (defined by the mean correct response rate of both creative chunks decomposition) showed significant and positive correlation with ReHo in the left AG/STG/IPL. Abbreviations: ReHo, regional homogeneity; STG, superior temporal gyrus; ACC, anterior cingulate cortex; CN, caudate nucleus; AG, angular gyrus; IPL, inferior parietal lobe.
